# 

**Published:** 1903-01-31

**Authors:** 


					&
the hospital. "The standard of highest purity.'?Lanset. jA*. 31st 1903
CADBURY'S ^ jjj ^ CADBURY'S
COCOA " 1 jr=^ JP COCOA
ABSOLUTELY PURE. NO DRUGS OR ALKALIES USED.
isoonr WesternbiFiauAay
No. 853: Vol. XXXIII. [IKS"] Saturday,
Jan. 31st, 1903.
SUbSCseePpi0^?ermj PRICH TWOPENCE.

				

